# Challenges in Diagnosing and Treating Acutely Febrile Children with Suspected Malaria at Health Care Facilities in the Lake Mwanza Region of Tanzania

**DOI:** 10.4269/ajtmh.23-0254

**Published:** 2023-12-26

**Authors:** Philip Koliopoulos, Neema Kayange, Christian Jensen, Britta Gröndahl, Jana Eichmann, Tim Daniel, Florian Huth, Till Eckert, Nele Klamm, Marlene Follmann, Grey Carolina Medina-Montaño, Adolfine Hokororo, Leah Pretsch, Julia Klüber, Christian Schmidt, Antke Züchner, Marylyn M. Addo, Bernard Okamo, Stephen E. Mshana, Stephan Gehring

**Affiliations:** ^1^ Center of Pediatric and Adolescent Medicine, University Medical Center, Mainz, Germany;; ^2^ Department of Pediatric and Adolescent Medicine, Bugando Medical Centre, Mwanza, Tanzania;; ^3^ Department of Pediatric and Adolescent Medicine, St. Joseph Hospital, Berlin, Germany;; ^4^ Department of Visceral and Thoracic Surgery, Klinikum Worms, Worms, Germany;; ^5^ Department of Internal Medicine, GeoMed Kreisklinik, Gerolzhofen, Germany;; ^6^ Center of Gynecology and Obstetrics, Augusta-Kranken-Anstalt, Bochum, Germany;; ^7^ Department of Internal Medicine, Gesundheits- und Pflegezentrum, Rüsselsheim, Germany;; ^8^ Department of Dermatology, University Medical Center, Mainz, Germany;; ^9^ Department of Infection and Immunity, Luxembourg Institute of Health, Esch-sur-Alzette, Luxembourg;; ^10^ Department of Pediatric and Adolescent Medicine, St. Vinzenz-Hospital, Dinslaken, Germany;; ^11^ CCBRT Maternity and Newborn Hospital, Dar es Salaam, Tanzania;; ^12^ Institute for Infection Research and Vaccine Development, University Medical Centre Hamburg-Eppendorf, Hamburg, Germany;; ^13^ Catholic University of Health and Allied Sciences, Mwanza, Tanzania

## Abstract

Acute febrile diseases transmitted by mosquitos are a diagnostic challenge for pediatricians working in sub-Saharan Africa. Misclassification due to the lack of rapid, reliable diagnostic tests leads to the overuse of antibiotics and antimalarials. Children presenting with acute fever and suspected of having malaria were examined at health care facilities in the Mwanza Region of Tanzania. The sensitivity and specificity of blood smear microscopy and malaria rapid diagnostic tests that targeted histidine-rich protein 2 and *Plasmodium* lactate dehydrogenase were compared with a multiplex reverse transcriptase-polymerase chain reaction (PCR)–ELISA. Six hundred ninety-eight children presented with acute fever and met the criteria for inclusion; 23% received antibiotics and 23% received antimalarials prior to admission. Subsequently, 20% were confirmed by PCR to have *Plasmodium falciparum* infection. Blood smear microscopy exhibited 33% sensitivity and 93% specificity. The malaria rapid test provided 87% sensitivity and 98% specificity in detecting acute malaria infections. Only 7% of malaria-negative children received antimalarials at Sengerema Designated District Hospital when treatment was guided by the results of rapid testing. In contrast, 75% of malaria-negative patients were treated with antimalarial drugs at health facilities that used blood smears as the standard diagnostic test. Misclassification and premedication of nonmalarial, febrile illnesses contribute to the emergence of antimalarial and antimicrobial resistance. The incorporation of malaria rapid diagnostic tests into the clinical routine translated into improved treatment and a significant reduction in antimalarial drug prescriptions.

## INTRODUCTION

The United Nations Children’s Fund reported that malaria is one of the major causes of mortality in children under the age of 5 years.^1^ According to the 2022 WHO World Malaria Report, an approximate 475,000 children in Africa died of malaria caused by *Plasmodium falciparum*.^2^ The Ministry of Health and Social Welfare in Tanzania addressed this problem in the 2018 Malaria Operational Plan and set goals to reduce mortality. Increased malaria awareness and the introduction of artemisinin-based combination therapy (ACT) have led to a tremendous decline in *P. falciparum* infections in Tanzania in the past.^3^ Unfortunately, global progress has stalled since 2017, and disruptions due to the COVID-19 pandemic have led to an increase in the numbers of malaria cases and deaths in the sub-Saharan (SSA) region.^4^

Blood smear microscopy is still considered the gold standard for detecting acute malaria infections.^5^ It can distinguish between *Plasmodium* spp., differentiate sexual and asexual stages, and score parasite density. Four out of five malaria cases identified by microscopy in southern Tanzania, however, were most likely false positives.^6^ Misclassification of malaria infections can be attributed, in part, to previous WHO recommendations for presumptive treatment of malaria in pediatric patients.^7^ The WHO malaria treatment policy was revised in 2012 to a “test and treat” strategy, requiring parasitological confirmation before administration of ACT.^8^ Health care workers at peripheral clinics still express uncertainty about treating pediatric patients diagnosed with malaria-negative, febrile diseases.^9^

Malaria rapid diagnostic tests (mRDTs) are reliable, easy tools that are currently used routinely in several countries in SSA.^10^ Their use significantly decreased the number of nonindicated prescription of antimalarial drugs in low-resource settings.^11^ The adherence of health care workers to mRDT results varies.^12^ Consequently, treatment of rapid test-negative patients with antimalarials in the health care centers of Ghana, Benin, and Tanzania is well documented.^12^^,^^13^ Additionally, health care workers experience difficulties differentiating viral from bacterial infections in declining malaria settings.^14^ Indeed, there is a growing concern over the unwarranted prescription of antibiotics to manage fevers unrelated to malaria in cases where the results of mRDTs are negative.^12^ Moreover, mRDTs are often optimized to detect *P. falciparum* and/or *Plasmodium vivax*, while the sensitivity to *Plasmodium malariae* and *Plasmodium ovale* is reduced.^15^ Accordingly, the prevalence of *P. malariae* and *P. ovale* may be significantly underestimated when relying solely upon mRDTs as a diagnostic tool.^16^ Furthermore, mutations in the genes that encode histidine-rich protein 2 (HRP2) and HRP3, which impact *Plasmodium* species detection by common mRDTs, are frequently reported.^17^^,^^18^

Artemisinin treatment, characterized by rapid parasite clearance, is the key therapeutic approach. This treatment, combined with slowly eliminated antimalarials aimed to delay the development of resistance, resulted in the highly effective 3-day-based ACT in the mid-1990s.^19^ The possible spread of artemisinin resistance to Africa represents a “disastrous” scenario, since new antimalarial drugs are not envisioned in the near future.^20^

To address these challenges, the use of highly sensitive and specific polymerase chain reaction (PCR)-based diagnostics is increasingly discussed.^15^^,^^21^ Polymerase chain reaction technology constitutes a valuable tool to properly assess disease burden and provides an opportunity to monitor resistance mutations.^22^ Despite its advantages, performing PCR is currently beyond the capabilities of many health care facilities in SSA. In this regard, dried blood spots offer a valuable method of transporting *P. falciparum*-positive samples. In a previous analysis of the present data, a high concordance between serum samples and a long-lasting preservation capability was documented.^23^

## MATERIALS AND METHODS

### Patient population and study sites.

A health care-based, cross-sectional prospective study was initiated at the Lake Victoria region, Tanzania. Pediatric patients were examined at three health care facilities: the Bugando Medical Center (BMC), the Sekou Toure Regional Referral Hospital (STRRH), and the Sengerema Designated District Hospital (SDDH). Sociodemographic data, clinical data, and blood samples were collected from 751 children (4 months to 17 years of age) between April 2016 and March 2018. Children were eligible for enrollment if they presented with acute high-grade fever (> 38.0°C) and met the criteria of presumptive malaria according to the WHO definition, i.e., presented with an illness suspected by local health care workers to be due to malaria with or without symptoms in addition to fever.^24^^,^^25^ The patients from BMC were hospitalized with presumptive malaria and routinely diagnosed by visual examination of blood smears. In contrast, the patients at STRRH and SDDH were seen at an outpatient department, and a malaria diagnosis was confirmed by mRDT. Notably, the mRDTs performed at STRRH took place after the patient was seen by a health care worker in a laboratory facility that was spatially separate from the examination room. At SDDH, the mRDT and clinical examination were performed in the same room. The current data were partially published elsewhere.^23^^,^^26^

### Sample collection and rapid diagnostic testing.

The protocol for sample collection was described previously.^23^^,^^26^ The NADAL^®^ Malaria Pf/Pan Ag 4 Species Test (nal von minden GmbH, Regensburg, Germany) is a lateral flow chromatographic immunoassay that detects HRP2 and *Plasmodium* lactate dehydrogenase, which are specific for *Plasmodium falciparum* and *Plasmodium* spp., respectively. The malaria rapid test was performed, and the results of additional testing, i.e., blood smear microscopy and mRDTs, were obtained at the health care facility indicated. The effect of the different diagnostic approaches to treating malaria was monitored by comparing the prescription rates of antimalarial and antibiotic medication at the three study sites. Whatman^®^ 903 protein sample cards spotted with patient whole blood were dried and prepared as described previously.^27^ The samples were shipped to the University of Mainz Department of Pediatric Immunology and Infectious Diseases according to International Air Transport Association (IATA) and International Civil Aviation Organization (ICAO) regulations. The sample card extraction procedure, including standardized punching, prevention of carryover contamination, and incubation with vortexing for 60 minutes at 72°C, was established by us.^23^

### MPCR–ELISA.

A multiplex reverse transcriptase-PCR (MPCR)–ELISA panel for malaria-like diseases was designed by the Laboratory of Pediatric Immunology and Infectious Diseases, Mainz, Germany.^23^ It covers nine mosquito-transmitted diseases and includes a highly sensitive primer set that targets *Plasmodium* species 18S rRNA genes (Table 1).^28^

**Table 1:** Diseases covered by MPCR-ELISA

Disease agents and family	Virus or bacterium
Arboviruses	
* Flaviviridae*	Dengue virus
	West Nile virus
	Zika virus
	Yellow fever virus
* Togaviridae* (alphaviruses)	Semliki Forest virus
	O’nyong’nyong virus
	Chikungunya virus
* Bunyaviridae*	Rift Valley fever virus
Protozoa	
* Plasmodiidae*	*P. falciparum*, *P. vivax*, *P. malariae*

MPCR = multiplex reverse transcriptase-polymerase chain reaction.

Total nucleic acid was isolated using the High Pure Viral Nucleic Acid Kit according to the manufacturer’s instructions (Roche Diagnostics, Mannheim, Germany). Subsequent amplification and gel electrophoresis were performed as described by Koliopoulos et al.^23^ The PCR product was then incubated with a 3′ biotinylated capture probe and anti-digoxigenin-peroxidase (Roche Diagnostics) on streptavidin-coated microtiter plates. Samples were classified based upon a cutoff value at an optical density at 405 nm (OD_405_) of 0.4; values between 0.2 and 0.4 were considered borderline, and these samples were retested using a singleplex PCR approach before classification. All *Plasmodium* species-positive samples were retested by trivalent PCR to differentiate between *P. falciparum*, *P. vivax*, and *P. malariae*.

### Biosafety.

All nucleic acid was isolated and extracted from pathogens in a biosafety level 2 cabinet in accordance with strict biosecurity standards. Each series of nine specimens included one negative control (NaCl) to monitor cross-contamination. Preparation of PCR reagents and nucleic acid extraction were conducted in different rooms. At the end of each procedure, Terralin^®^ (Schülke & Mayr GmbH, Norderstedt, Germany) liquid and ultraviolet light (30 minutes) were used to eliminate contamination.

### Statistical analysis.

The sensitivity and specificity of malaria diagnostic tests (i.e., blood smear microscopy versus mRDT) were evaluated using two-by-two tables comparing the results of each test with those obtained by MPCR-ELISA. The 95% CIs were calculated according to the Clopper-Pearson method. The data were analyzed with two statistical programs, SPSS 25 (IBM SPS Statistics, Armonk, NY) and Sigma Plot 11 (Systat Software GmbH, Erkrath, Germany).

## RESULTS

### Study population and sociodemographic data.

Data obtained from 751 pediatric patients were evaluated. Six hundred ninety-eight of these patients were between 4 months and 17 years of age and met the inclusion criteria by presenting with an acute fever of > 38.0°C (Table 2). One hundred twenty of the children enrolled in the study were from the general pediatric ward at BMC, 310 were from the pediatric outpatient department at STRRH, and 268 were from the pediatric outpatient department at SDDH. The gender distributions were similar at all study sites, with a higher percentage of male than female patients. The mean age and body temperature were 20 months and 38.7°C, respectively. Children at SDDH were slightly younger than those at the other study sites.

**Table 2:** Study populations enrolled at three study sites

Site	Number of patients	Gender	Median age (range)	Median temperature (range)	Period
BMC	120	M **=** 63 (52%), F **=** 57 (48%)	26 months (5–144 months)	38.7°C (38.0–41.0°C)	April 2016–October 2016
STTRH	310	M **=** 184 (59%), F **=** 126 (41%)	21 months (4–144 months)	38.5°C (38.0–41.0°C)	September 2016–March 2018
SDDH	268	M **=** 154 (58%), F **=** 114 (42%)	17 months (4–108 months)	39.0°C (38.0–41.5°C)	March 2017–July 2018
Average	698	M **=** 401 (57%), F **=** 297 (43%)	20 months (4–144 months)	38.7°C (38.0–41.5°C)	April 2016–March 2018

BMC = Bugando Medical Centre; F = female; M = male; SDDH = Sengerema Designated District Hospital; STTRH = Sekou Toure Regional Referral Hospital.

### Diagnostics.

MPCR-ELISA was performed on blood samples obtained from all 698 patients. A positive test for either *Plasmodium* spp. or *P. falciparum* was confirmed for 137 samples; the prevalences were similar at all study sites (Table 3). *Plasmodium* species PCR-positive children were older than the PCR-negative children; gender distribution and body temperature were similar (Table 4). No evidence of arbovirus infection was found in any of the samples.

**Table 3:** *Plasmodium* species PCR testing and prevalence of malaria at three study sites

Results	BMC	STRRH	SDDH	Average
PCR positive	26/120	56/310	55/268	137/698
Prevalence*	22% (15–30%)	18% (14–23%)	21% (16–26%)	20% (17–23%)

BMC = Bugando Medical Centre; F = female; M = male; PCR = polymerase chain reaction; SDDH = Sengerema Designated District Hospital; STTRH = Sekou Toure Regional Referral Hospital.

* Prevalence was calculated based upon *Plasmodium* species PCR results. The 95% CI, calculated according to the Clopper-Pearson exact method, is given in parentheses.

**Table 4:** Demographic data according to *Plasmodium* species PCR test results

Test result	Number of patients	Gender	Median age (min–max)	Median temperature (min–max)
PCR positive	137/698 (20%)	M = 56% (77/137), F = 44% (60/137)	35 months (4–120 months)	38.8°C (38.0–41.5°C)
PCR negative	561/698 (80%)	M = 58% (324/561), F = 42% (237/561)	16 months (4–144 months)	38.9°C (38.0–41.1°C)

F = female; M = male; max = maximum; min = minimum; PCR = polymerase chain reaction.

### Comparison of malaria diagnostics.

The accuracy of malaria diagnostic tests available locally was evaluated. At BMC, 112/120 (93%) of the patients were evaluated by blood smear microscopy; 14 tested positive. At SDDH, on the other hand, 123/268 subjects (46%) were tested using CareStart^™^ mRDT (Access Bio, Somerset, NJ); 37 patients tested positive. At STRRH, 270/310 (87%) patients were tested using mRDTs available locally (unknown manufacturer); 36 tested positive. Six hundred fifty-eight patients were retested by study personnel using the NADAL mRDT (nal von minden GmbH, Regensburg, Germany); a positive result was obtained for 125.

Sensitivity and specificity, calculated for mRDT and blood smear microscopy, were standardized in terms of the results of PCR. The NADAL mRDT exhibited 87% sensitivity and 98% specificity (Table 5). The positive and negative predictive values were 93% and 97%, respectively. Similarly, locally available mRDT showed high specificity at STRRH and SDDH; sensitivity varied. Blood smear analysis performed at BMC was the least accurate, revealing a specificity equivalent to that of the others but a much lower sensitivity of 33%.

**Table 5:** Sensitivity and specificity of malaria tests used clinically*

Performance	NADAL mRDT (*n =* 658)	Local mRDT† at STRRH (*n =* 270)	CareStart mRDT at SDDH (*n =* 123)	Blood smear at BMC (*n =* 112)
Sensitivity	87% (116/134) [80–92%]	74% (35/47) [60–86%]	97% (33/34) [85–100%]	33% (8/24) [16–55%]
Specificity	98% (515/524) [97–99%]	100% (222/223) [98–100%]	96% (85/89) [89–99%]	93% (82/88) [86–97%]

BMC = Bugando Medical Centre; mRDT = malaria rapid diagnostic test; SDDH = Sengerema Designated District Hospital; STTRH = Sekou Toure Regional Referral Hospital.

* The results obtained by malaria tests used clinically (blood smear microscopy and mRDTs) for acutely febrile children were compared with those obtained for the same patient that tested positive by multiplex reverse transcriptase-polymerase chain reaction–ELISA. Concordance for positive test results is given in parentheses. The 95% CIs according to the Clopper-Pearson exact method are given in square brackets.

† mRDT manufacturer unknown.

### Medication.

History of pharmacological treatment was obtained from medical records. Antipyretics and antibiotics were prescribed for an average 85% and 79% of subjects, respectively (Table 6). Nearly one-fourth of patients received a combination of several antibiotics. Antimalarial medication was prescribed to an average of 39% of patients. Prescription rates of both antimalarials and antibiotics were higher at BMC than at either STRRH or SDDH.

**Table 6:** Prescription rates at different study sites

Medication	BMC	STRRH	SDDH	Average
Antipyretics	81% (88/108)	74% (216/290)	98% (257/263)	85% (561/661)
Antibiotics	92% (99/108)	83% (241/290)	70% (186/265)	79% (526/663)
Antimalarials	81% (87/108)	35% (101/290)	27% (70/263)	39% (258/661)

BMC = Bugando Medical Centre; SDDH = Sengerema Designated District Hospital; STTRH = Sekou Toure Regional Referral Hospital.

Ninety-one percent (120/132) of PCR-positive patients and 26% (138/529) of PCR-negative patients were treated with antimalarial drugs. Antibiotics, on the other hand, were prescribed to 49% (65/123) of PCR-positive and 87% (461/531) of PCR-negative patients. The antipyretic prescription rates were similar in both groups, 86% and 85% respectively.

The rate of antimalarial prescriptions for malaria PCR-positive and -negative patients was calculated at each study site. Greater prescription rates (100%) for malaria PCR-positive pediatric patients at both BMC and SDDH were found than for patients treated with antimalarials at STRRH, at 77%. At BMC, however, 63/83 (75%) of malaria PCR-negative patients were prescribed antimalarial drugs. Consequently, antimalarials were most accurately prescribed at SDDH, where all PCR-positive patients were treated and treatment of 15/208 (7%) PCR-negative patients was relatively low.

### Clinical presentation and medical history.

Medical history was obtained by a caregiver from all 698 eligible patients. Additionally, all patients received a physical exam administered by trained study personnel. More than half of the patients reported respiratory and gastrointestinal symptoms (Table 7). Other common clinical findings included pallor and pain, hepato- or splenomegaly, and signs of central nervous system (CNS) infection. Rash, dehydration, and dysuria were reported less frequently. Prevalence of clinical features varied between study sites. Clinical features of severe malaria, including signs of cerebral infection, pallor, and bleeding, were noted more frequently in patients presenting at the tertiary health care facility, BMC. Pallor, the most common feature in patients who tested PCR positive, was nearly 2-fold less prevalent in PCR-negative patients. Similarly, hepatomegaly, splenomegaly, and/or signs of cerebral infection were noted much more frequently in patients who tested PCR positive. Respiratory symptoms, on the other hand, were more frequently observed in PCR-negative patients.

**Table 7:** Clinical features of patients at sites divided according to *P. falciparum* PCR test results

Clinical feature	BMC	STRRH	SDDH	Average	*P. falciparum*	
					PCR+	PCR−
Respiratory symptoms	53% (63/120)	74% (228/310)	43% (115/268)	58% (406/698)	49% (67/137)	60% (339/561)
Gastrointestinal symptoms	64% (77/120)	62% (191/310)	44% (119/268)	55% (387/698)	60% (82/137)	54% (305/561)
Pallor	51% (61/120)	45% (138/310)	25% (67/268)	38% (266/698)	62% (85/137)	32% (181/561)
Pain	56% (66/119)	28% (86/310)	28% (74/268)	32% (226/697)	40% (55/137)	31% (171/560)
Hepato- and/or splenomegaly	29% (35/119)	14% (42/309)	21% (56/265)	19% (133/693)	45% (61/136)	13% (72/557)
Signs of cerebral infection	28% (34/120)	15% (47/310)	6% (17/268)	14% (98/698)	28% (39/137)	11% (59/561)
Rash	16% (19/120)	9% (27/310)	11% (29/268)	11% (75/698)	9% (12/137)	11% (63/561)
Dehydration	7% (8/120)	11% (34/310)	8% (21/268)	9% (63/698)	9% (13/137)	9% (50/561)
Signs of urinary tract infection	13% (16/120)	7% (14/205)	9% (23/268)	9% (53/593)	17% (21/124)	7% (32/469)
Edema	19% (23/120)	5% (14/310)	2% (5/268)	6% (42/698)	9% (13/137)	5% (29/561)
Signs of bleeding	16% (19/120)	4% (12/309)	2% (2/133)	6% (33/562)	9% (9/104)	5% (24/458)

BMC = Bugando Medical Centre; PCR = polymerase chain reaction; PCR+ = PCR positive; PCR− = PCR negative; SDDH = Sengerema Designated District Hospital; STTRH = Sekou Toure Regional Referral Hospital.

### Premedication.

Premedication details were obtained from 97% and 100% of the patients before presentation at STRRH and SDDH, respectively. Available data at BMC were limited. Antipyretic treatment was reported by 49% of patients; use of antimalarials and antibiotics was noted in approximately half that percentage. The rates of antibiotic premedication were similar at STRRH and SDDH, with 65/399 (22%) and 64/267 (24%) patients, respectively. Use of antipyretics was more common in patients presenting at SDDH than at STRRH.

Premedication rates were compared in terms of *Plasmodium* species PCR testing results. Pretreatment with antibiotics was similar for both positive and negative groups, with 21% (23/110) and 23% (106/457), respectively. Notably, a greater percentage of PCR-positive patients, with 32% (35/110), than PCR-negative patients, with 20% (93/457), were premedicated with antimalarials.

### Seasonality.

The prevalence of malaria varied seasonally, suggesting that prescription rates might also vary between seasons. The entire dry season comprises 6 months per year, namely, January, February, and June to September; the long rainy season is March to May, and the short rainy season is October to December. A greater number of patients, 301/698 (43%), was enrolled during the dry season than during the short or long rainy seasons, in which 168/698 (24%) and 229/698 (33%) were enrolled, respectively. Notably, the prevalence of malaria was highest during the dry season, at 22% (66/301), and lowest during the short rainy season, at 15% (26/168). Concordantly, the antimalarial treatment rate of patients during the dry season, at 45% (129/286), was higher than the treatment rate during either the short or long rainy season, at 37% (55/150) and 33% (74/225), respectively.

## DISCUSSION

In the present study, 20% of pediatric patients presenting with acute febrile illness at health care facilities in the Mwanza region of Tanzania were *P. falciparum* PCR positive. Pallor, hepatosplenomegaly, and signs of CNS infection were more likely to occur in children with confirmed parasitemia. Fever studies conducted on children from mainland Tanzania over the last 15 years showed a huge variation in the prevalence of malaria and inconsistent results that varied depending upon the testing methods (see Supplemental Table 1). Large malaria prevalence studies conducted among 49,113 school children in Tanzania in 2014–2015 also documented substantial variation across regional and subregional levels.^29^

Although examination of Giemsa-stained thick blood smear remains the standard diagnostic test for acute malaria infection in many areas, studies including the sensitivity data presented herein document a high rate of misclassification.^30^^,^^31^ Difficulties identifying low-density parasitemia and dependency on the examiner’s skill pose major limitations to the blood smear method.^32^^,^^33^

Alternatively, mRDTs are easily used and provide fast and reliable results. The rate of mRDT testing for suspected malaria cases in SSA rose from 36% in 2010 to 84% in 2018.^34^ However, an increasing number of mRDT manufacturers has resulted in variability in testing performance. Meta-analysis conducted in 2022 concluded that conventional mRDTs might be only 42% sensitive.^35^ Consequently, the WHO initiated a laboratory evaluation program to provide comparative data of test performances.^36^ The mRDTs examined in the current study exhibited high specificity but only moderate (∼85%) sensitivity. The moderate sensitivity of mRDTs targeting HRP2 and *Plasmodium* lactate dehydrogenase to detect *P. falciparum* might be due to asymptomatic parasitemia, a known phenomenon in areas where malaria is endemic.^37^ The detection limit of conventional mRDTs is only comparable to blood smear microscopy, i.e., 100 to 200 parasites/µL. Moreover, mutations in the genes that encode the targeted *Plasmodium* species epitopes HRP2 and HRP3 exert a dramatic effect on the sensitivity of common mRDTs.^17^^,^^38^ In addition, gametocytemia, cross-reactivity (e.g., *Salmonella typhi*), and persistent positivity after antimalarial treatment are potential sources of false mRDT-positive results.^39^ Consequently, mRDTs should not be relied upon solely to judge the elimination of malaria.^32^^,^^36^^,^^40^

Polymerase chain reaction-based techniques offer a highly sensitive and specific approach to diagnosing malaria, especially in cases of low parasitemia.^41^ However, molecular methods including conventional PCR, quantitative PCR, and multiplex PCR are time-consuming, require expensive reagents, and demand trained personnel; as such, these methods have limited value in low-resource settings.^42^ Consequently, MPCR-based diagnosis of mosquito-borne diseases in patients with suspected malaria remains beyond the current capacity of low-level health care facilities in the Lake Mwanza region of Tanzania. WHO summed up the requirements for point-of-care testing, which targeted *P. falciparum* in the acronym ASSURED (affordable, sensitive, specific, user-friendly, rapid and robust, equipment-free, and deliverable to end users).^43^

Recent studies conducted on untreated febrile children in Dar es Salaam, Tanzania, suggest that low-density parasitemia detected by highly sensitive PCR diagnostic tests is self-resolving and might not need antimalarial therapy.^44^ In such cases, it is conceivable that a nonmalarial infection rather than *P. falciparum* is the underlying cause of fever.^45^ Epidemiologists focusing on non-malaria-related, febrile illnesses are discussing alternative approaches to quantifying the parasite burden. Statistical models estimate that only 38% of *P. falciparum*-positive fevers can be actually attributed to malaria, suggesting a high rate of undetected coinfections, which were not investigated in the present investigation.^45^

Antimalarial and antibiotic medications are readily accessible in SSA. Nearly one-fourth of the acute, febrile patients reported premedication in the current study. A higher percentage of *P. falciparum* PCR-positive than PCR-negative patients received antimalarials. The wide practice of self-medication is a serious concern.^46^ The prescription rates of antimalarials and antibiotics in the current study are worrisome. The difference in severity and complexity of pediatric cases must be considered when comparing prescription rates. This is evidenced by clinical features such as symptoms of CNS infection or signs of bleeding, which occurred more frequently at BMC (a tertiary health care facility) than at the STRRH or SDDH study sites (secondary-primary facilities).

The fraction of *P. falciparum*-negative patients receiving antimalarials was 10 times lower at SDDH than at BMC. The established use of mRDTs was a significant factor contributing to this reduction; tests at SDDH were performed routinely in examination rooms before medications were prescribed. In contrast, microscopic examination of blood smears in the laboratory department was the standard diagnostic method at BMC. A study conducted in northeastern Tanzania did not find that the overprescription of antimalarials was driven by patient pressure.^47^ Rather, effective communication of test results was encouraged to improve prescribing practices. Reduced nonindicated treatment in clinical practice requires that health care workers trust negative mRDT results.^9^ Antimalarial prescription rates ranging up to 42% in low-transmission SSA settings document a serious health threat for vulnerable pediatric patients.^48^

In an analysis of the medications prescribed to 522,480 mRDT-tested patients with acute fever, Hopkins et al. concluded that the implementation of mRDTs might increase the untargeted prescription of antibiotics.^49^ This conclusion correlates with studies evaluating a census of national health care facilities in Malawi, which demonstrated a 16.8-times higher rate of antibiotic overtreatment of mRDT-negative than of mRDT-positive patients.^50^ Data derived from the Lake Victoria region in the current study revealed that antibiotics were prescribed to 86% and 46% of mRDT-negative and mRDT-positive patients, respectively. These results demonstrate a reduction in unwarranted antibiotic prescriptions in confirmed cases of malaria.

Moreover, clinical decision algorithms on mobile systems for the integrated management of childhood illnesses (e.g., electronic integrated management of childhood illness [e-IMCI] or algorithm for the management of childhood illness [ALMANACH]) have the proven ability to reduce antibiotic prescriptions up to 80% by properly addressing the treatment of mRDT-negative cases.^51^^,^^52^

In conclusion, the factors enumerated above emphasize the complex challenges for health care workers in SSA to strictly follow the “test, treat, and track” recommendation of the WHO.^53^ The data indicate that the diagnostic approach should consider the level of health care available in malaria-endemic countries. Primary and secondary health care facilities should implement cost-effective mRDTs to diagnose all children with acute febrile illness. A diagnosis of malaria in tertiary health care facilities treating clinically severe pediatric cases could be supported by a PCR confirmation test.^54^ Establishing the technical and human capacity to perform PCR-based malaria diagnostics is mandatory for the future response to challenges, such as the rise of artemisinin resistance. Only then can the global aim to prevent “potentially untreatable falciparum malaria” be achieved.^55^ Additionally, an awareness of nonmalaria febrile infections and affordable and reliable point-of-care testing (host biomarkers and relevant pathogens) supported at all health care levels by mobile decision algorithms are the key to reducing the incidences of nonindicated treatment and the emergence of critical drug-resistant mutants.

## Supplemental files

10.4269/ajtmh.23-0254 Supplemental Materials
