# Supplementary material for: Challenges in Diagnosing and Treating Acutely Febrile Children with Suspected Malaria at Health Care Facilities in the Lake Mwanza Region of Tanzania
Source: Am J Trop Med Hyg. 2023 Dec 26;110(2):202–8. doi: 10.4269/ajtmh.23-0254 (PMC10859794; doi:10.4269/ajtmh.23-0254)
Supplement: Supplemental Materials [file tpmd230254.SD1.pdf]

SUPPLEMENTARY INFORMATION

Table S1. Prevalence of malaria reported in recent studies of fever conducted on mainland Tanzania

Study[a]	Study setting	Malaria test and results
**Crump *et al.* (2007-2008)[1]**	n = 467 (age 2 months - 13 years), Moshi, acute fever/history of fever < 48h	1% (6/467); blood smear
**D'Acremont *et al.* (2008)[2]**	n = 1005 (age 2 months - 10 years), Ifakara and Dar es Salaam, acute fever > 38.0°C	11% (105/1005); blood smear or mRDT
**Mahende *et al.* (2009-2010)[3]**	n = 867 (age 2 – 59 months), Korogwe District, fever/history of fever < 48h	8% (72/867); blood smear<br>10% (85/867); mRDT<br>12 % (79/677); PCR
**Mazigo *et al.* (2010)[4]**	n = 300 (age 2 – 59 months), Nzega District, Tabora, acute fever/ history of fever < 2weeks	12% (36/300); blood smear
**Koliopoulos, Kayange *et al.* (2016-2018)**	n = 698 (age 4 month – 12 years), Mwanza Region , acute fever	13% (14/112); blood smear<br>19% (125/658); mRDT<br>20% (137/698); PCR
**Chipwaza *et al.* (2013)[5]**	n = 370 (age 2 - 13 years), Kilosa district, Morogoro region, acute fever	24% (144/609); blood smear
**Strøm *et al.* (2009)[6]**	n = 304 (age 1 month - 7 years), Dar es Salaam, acute fever	13% (40/304); blood smear<br>12% (36/304); mRDT<br>25% (76/304); PCR
**Nkonya *et al.* (2014)[7]**	n = 600 (age 2 months - 13 years), Misungwi district, Mwanza region, acute fever/history of fever < 48h	17% (102/600); blood smear<br>33% (197/600); mRDT
**Mtove *et al.* (2006-2007)[8]**	n = 3639 (age 2 months - 13 years), Muheza, northeast Tanzania, acute fever/history of fever < 48h	60% (2195/3639); blood smear<br>73% (2640/3639); mRDT

[a]Period of sample collection in parentheses.

REFERENCES

1. Crump JA, Morrissey AB, Nicholson WL, Massung RF, Stoddard RA, Galloway RL, Ooi EE, Maro VP, Saganda W, Kinabo GD, Muiruri C, Bartlett JA, 2013. Etiology of severe non-malaria febrile illness in Northern Tanzania: a prospective cohort study. PLoS Negl Trop Dis 7: e2324.
2. D'Acremont V, Kilowoko M, Kyungu E, Philipina S, Sangu W, Kahama-Maro J, Lengeler C, Cherpillod P, Kaiser L, Genton B, 2014. Beyond malaria--causes of fever in outpatient Tanzanian children. N Engl J Med 370: 809-17.
3. Mahende C, Ngasala B, Lusingu J, Yong TS, Lushino P, Lemnge M, Mmbando B, Premji Z, 2016. Performance of rapid diagnostic test, blood-film microscopy and PCR for the diagnosis of malaria infection among febrile children from Korogwe District, Tanzania. Malar J 15: 391.
4. Mazigo HD, Meza W, Ambrose EE, Kidenya BR, Kweka EJ, 2011. Confirmed malaria cases among children under five with fever and history of fever in rural western Tanzania. BMC Res Notes 4: 359.
5. Chipwaza B, Sumaye RD, 2020. High malaria parasitemia among outpatient febrile children in low endemic area, East-Central Tanzania in 2013. BMC Res Notes 13: 251.
6. Strom GE, Haanshuus CG, Fataki M, Langeland N, Blomberg B, 2013. Challenges in diagnosing paediatric malaria in Dar es Salaam, Tanzania. Malar J 12: 228.
7. Nkonya DN, Tarimo DS, Kishimba RS, 2016. Accuracy of clinical diagnosis and malaria rapid diagnostic test and its influence on the management of children with fever under reduced malaria burden in Misungwi district, Mwanza Tanzania. Pan Afr Med J 25: 48.
8. Mtove G, Nadjm B, Amos B, Hendriksen IC, Muro F, Reyburn H, 2011. Use of an HRP2-based rapid diagnostic test to guide treatment of children admitted to hospital in a malaria-endemic area of north-east Tanzania. Trop Med Int Health 16: 545-50.
